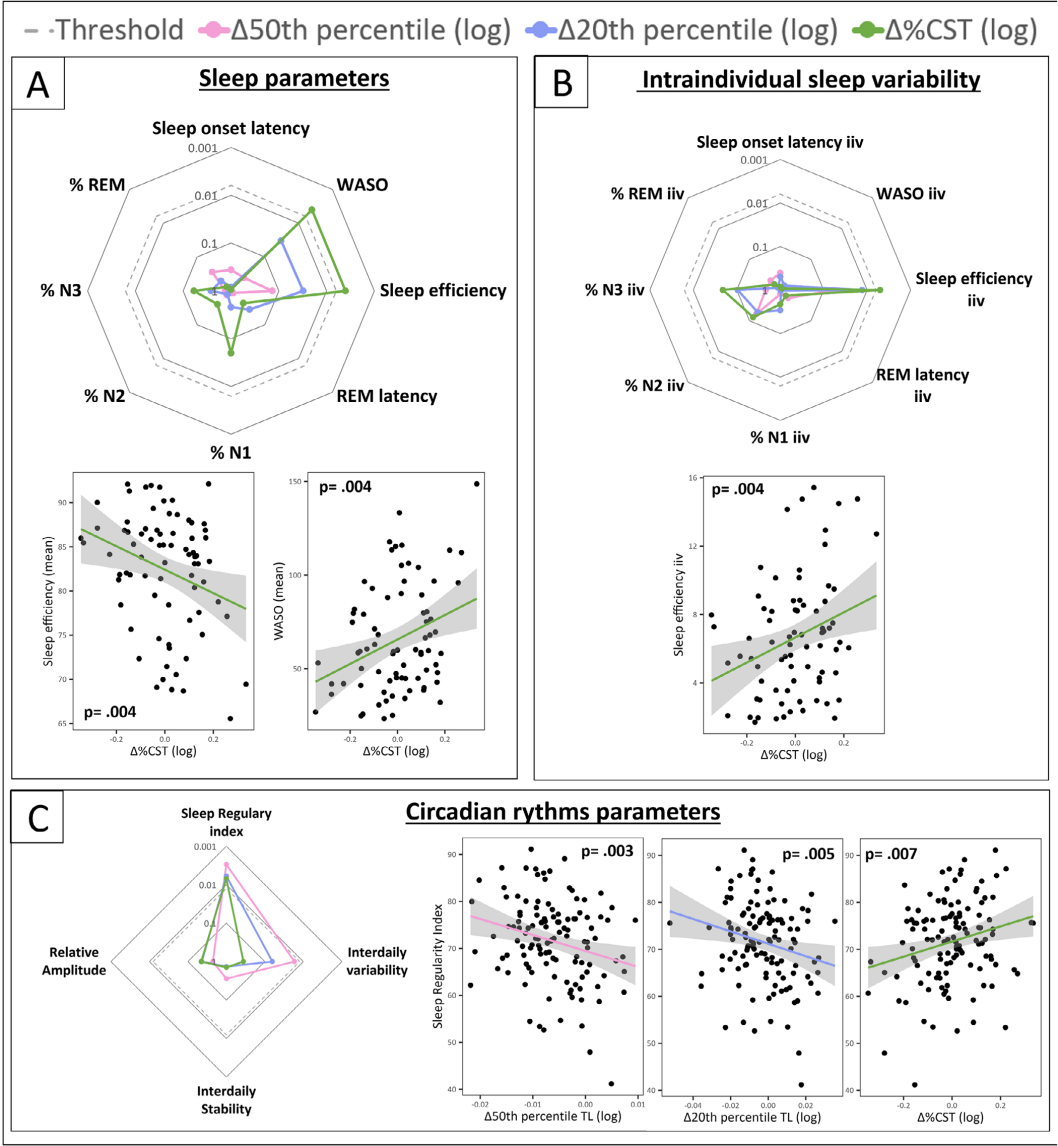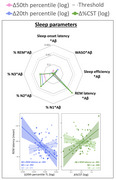# Telomere Dynamics Influenced by Sleep, Sleep Variability and Circadian Rhythms in Older Adults With and Without Alzheimer's Risk

**DOI:** 10.1002/alz70856_104780

**Published:** 2026-01-07

**Authors:** Asrar Lehodey, Blandine Montagne, Stéphane Rehel, Perla Kaliman, Anne Chocat, Florence Mézenge, Brigitte Landeau, Denis Vivien, Vincent De la Sayette, Gael Chételat, Géraldine Rauchs, Géraldine Poisnel, the Medit‐Ageing Research Group

**Affiliations:** ^1^ Inserm, Inserm UMR‐S U1237, Université de Caen‐Normandie, GIP Cyceron, Boulevard H. Becquerel, Caen, France; ^2^ Faculty of Health Sciences, University Oberta de Catalunya, Barcelona, Spain; ^3^ Département de Recherche Clinique, CHU Caen‐Normandie, Caen, France; ^4^ Service de Neurologie, CHU Caen‐Normandie, Caen, France

## Abstract

**Background:**

Sleep and circadian rhythms disturbances have been linked to cognitive decline and an increased risk of Alzheimer's disease (AD). These disruptions are also closely associated with biological aging processes. Telomere shortening, a key marker of cellular aging, has been implicated in various age‐related diseases, including AD. Although sleep disturbances have been linked to shorter telomere length (TL), the effects of sleep, its variability, and circadian rhythms on telomere dynamics—particularly the percentage of critically short telomeres (%CST), which would be a more specific marker of brain aging and vulnerability to AD—remains unknown. Furthermore, the interplay between these factors and AD risk has yet to be investigated in healthy older adults.

**Method:**

Data from 124 healthy older adults (mean age ± SD: 69.27 ± 3.73y) from the Age‐Well interventional trial (NCT02977819) were analyzed. Amyloid (Aβ) status was assessed using AV45‐PET, and blood samples were collected to determine three TL measures (50th and 20th percentile TL, and %CST) at baseline and after an 18‐month follow‐up. Sleep and its variability were assessed using the Somno‐Art® device (5 nights), and circadian rhythms with actigraphy (1 week). Multiple linear regressions examined whether baseline sleep and circadian rhythms‐related measures predicted TL changes over time. Interaction analyses were performed to examine the modulatory effects of Aβ and APOE4 status on these relationships. Age, sex, education, BMI, and intervention group were included as covariates. Only associations surviving Bonferroni correction are reported.

**Result:**

Poor sleep quality (lower sleep efficiency and higher WASO) and greater variability in sleep efficiency predicted an increase in %CST (Figure 1A, 1B). Greater regularity in sleep/wake patterns was associated with a decrease in 50th and 20th percentile TL and an increase in %CST (Figure 1C). In Aβ‐positive individuals, longer rapid eye movement sleep latency predicted a reduction in 20^th^ percentile TL and an increase in %CST (Figure 2).

**Conclusion:**

This study suggests that sleep, its variability and circadian rhythms may accelerate cellular aging through telomere shortening in older adults with and without AD risk factors. Our results highlight the potential value of interventions targeting sleep to reduce biological aging and vulnerability to age‐related diseases.